# Subjective Perceptions of Digital Phenotyping Measures of Negative Symptoms Among Individuals At Clinical High Risk for Psychosis and Their Relatives

**DOI:** 10.1093/schizbullopen/sgag034

**Published:** 2026-08-18

**Authors:** Zhixin Zhang, Ada L Hutcheson, Lauren E Arnold, Zachary A Carter, Justin T Barolette, Josephine E Luck, Kimberly Kundert-Obando, Anna R Knippenberg, Elaine F Walker, Vijay A Mittal, Gregory P Strauss

**Affiliations:** Department of Psychology, University of Georgia, Athens, GA 30602, United States; Department of Psychology, University of Georgia, Athens, GA 30602, United States; Department of Psychology, University of Georgia, Athens, GA 30602, United States; Department of Psychology, University of Georgia, Athens, GA 30602, United States; Department of Psychology, University of Georgia, Athens, GA 30602, United States; Department of Psychology, University of Georgia, Athens, GA 30602, United States; Department of Psychology, University of Georgia, Athens, GA 30602, United States; Department of Psychology, University of Georgia, Athens, GA 30602, United States; Department of Psychology and Program in Neuroscience, Emory University, Atlanta, GA 30322, United States; Department of Psychology, Northwestern University, Evanston, IL 60208, United States; Department of Psychology, University of Georgia, Athens, GA 30602, United States

**Keywords:** clinical high risk, digital phenotyping, qualitative

## Abstract

**Objective:**

Negative symptoms are frequently experienced among youth at clinical high-risk (CHR) for psychosis. While clinical interviews are the most well-established and widely used measurements for assessing negative symptoms in individuals at CHR, several limitations exist. Digital phenotyping methods (eg, geolocation, ecological momentary assessment [EMA]) have shown promise in measuring negative symptoms across contexts. However, little is known about how CHR and their relatives (REL) perceive these methods or understand EMA questions. Accounting for subjective viewpoints is important before these tools can be utilized at scale and in clinical trials.

**Method:**

Groups of CHR (*n* = 45) and REL (*n* = 18) participants completed structured surveys and qualitative interviews evaluating perceived importance of and concerns regarding digital phenotyping measures to assess negative symptoms as well as the perceived clarity of EMA survey questions.

**Results:**

Both groups showed low levels of concern regarding digital phenotyping methods. Concerns mainly revolved around privacy and data security, and were mitigated after clarifications on protection policies and research procedures were provided. Participants reported that active and passive digital phenotyping measurements were moderately important and could accurately measure their symptoms. They also deemed most EMA questions clear and provided suggestions for revising those they found less clear.

**Conclusions:**

Clinical high-risk and REL participants largely supported the use of digital phenotyping to measure negative symptoms, endorsing its perceived relevance and practical feasibility. These findings contribute to ongoing efforts to align clinical assessments tools with lived experience and to provide support for regulatory standards for patient-centered outcome measures.

## Introduction

Schizophrenia (SZ) is sometimes characterized by a prodromal (ie, pre-illness) period where individuals experience attenuated positive symptoms (ie, reduced conviction, frequency) and functional decline beginning approximately 2-4 years prior to illness onset.^1^ This period, when individuals are at clinical high-risk (CHR) for developing psychosis represents a critical window for early identification and prevention. Negative symptoms (ie, anhedonia, avolition, asociality, blunted affect, alogia) are frequently experienced among individuals at CHR and are one of the strongest predictors of conversion to full psychosis and functional decline,^2^^,^^3^ paralleling their important prognostic status in individuals with SZ.^4^ As such, being able to accurately assess and monitor changes in negative symptoms over time is critical for early identification and prevention among individuals at CHR.

Currently, the gold standard for assessing negative symptoms in CHR relies on clinician-rated interviews.^5^^,^^6^ These measures require interviewers to evaluate whether CHR experience reductions in normative aspects of emotional experience, motivation, speech, social behavior, and emotional expression based on participant self-report and observation during the interview period. Early measures, such as the Structured Interview for Psychosis-Risk (SIPS) and the Comprehensive Assessment of At-Risk Mental States (CAARMS), which have negative symptom subscales, had issues which limited their reliability and validity.^6^ However, more recent second-generation measures, such as the Brief Negative Symptom Scale (BNSS),^7^ Clinical Assessment Interview for Negative Symptoms (CAINS),^8^ and The Negative Symptom Inventory-Psychosis Risk (NSI-PR)^9^ offer more modern conceptualizations and psychometrically validated tools. These scales have allowed clinicians and researchers to monitor negative symptom changes longitudinally in CHR to predict long-term clinical outcomes effectively (eg, conversion, remission, progression, persistence). Although clinical interviews serve an indispensable and key role in negative symptom assessment, they have several known limitations inherent to the interview method itself (eg, limited ecological validity, prone to self-report biases, contextually and temporally insensitive).^1^

Recent technological developments now make it possible to overcome some of the limitations associated with clinical interviews for negative symptoms. The broad umbrella term for this approach is *digital phenotyping*, which refers to collecting data in daily life through smartphones and smart bands.^10–12^ Digital phenotyping is typically divided into active (ie, requires actively performing data collection behaviors) and passive (ie, without active data collection behaviors) measurements. Examples of active measures include: ecological momentary assessment^13–16^ (EMA; requires response to a brief questionnaire several times a day), ambulatory video or audio recordings^17^^,^^18^ (requires participants to take a video/audio recording of themselves and respond to specific prompts). Examples of passive measures include: accelerometry^14^^,^^19^^,^^20^ (vigor and variability of motor movements), geolocation^21^^,^^22^ (collection of GPS coordinates to monitor movement patterns), vocal acoustic activity (VOX; the level of human speech detected amidst ambient background noise),^23^^,^^24^ and turn latency (the length of time between the end of one speaker’s response and the time it takes the participant to respond). These tools have been receiving increased attention as methods for assessing negative symptoms in individuals with schizophrenia, and there is support for their reliability and validity as negative symptom assessments.^13^^,^^14^^,^^17^^,^^22^^,^^25^

Despite the increasing popularity of using digital phenotyping to measure negative symptoms, several important questions need to be addressed before these tools can be used as primary outcome measures. Primary aims of the current study were to determine how individuals at CHR and their relatives (REL) subjectively view these assessments, what concerns they have with their use, how well they understand the EMA questions, and whether they think digital technologies can accurately assess their symptoms and predict important outcomes such as functioning and quality of life. Understanding these perspectives could facilitate the psychometric validation of digital phenotyping assessments, improve them to increase adherence rate, inform data interpretation, and how to use these technologies as outcome measures in clinical trials and routine clinical monitoring (eg, by incorporating consumer views on factors influencing adherence and expectations regarding data privacy). To resolve this gap in knowledge, the current study obtained qualitative and quantitative subject impressions of digital phenotyping assessments in a sample of CHR participants and their REL. Since the specific aim of the study was to obtain qualitative and quantitative descriptions of digital phenotyping measures, specific hypotheses were not made.

## Methods

### Participants

Two groups of participants were recruited in the study: (1) individuals at CHR for psychosis (*n* = 45); (2) relatives (REL; *n* = 18), who were parents, siblings, or spouses who had known the participant for several years. On average relatives had known the CHR participant for 17.67 years (SD = 10.92): parents = 29.25 (SD = 6.75); siblings = 22.71 (SD = 4.27); and spouses = 6.00 (SD = 4.32).

Prior studies indicate that a portion of individuals at CHR demonstrate limited insight and this profile is associated with higher negative symptoms.^26^^,^^27^ Given that collateral informant reports can be helpful for overcoming the impact of insight on self-report data, relatives were also recruited to obtain their unique perspective and determine whether qualitative findings differed fundamentally from the CHR group. Because of some aspects of negative symptoms are behavioral, and individuals can sometimes have less insight into the absence of such behaviors, collateral informants may be useful to understand negative symptoms.

Clinical high-risk participants who participated in prior studies in our lab were invited to complete the current study. The studies that participants were initially recruited to complete focused on reward processing, emotion regulation, or negative symptom assessment in CHR.^9^^,^^28–32^ Clinical high-risk participants were recruited from the Georgia Psychiatric Risk Evaluation Program (G-PREP) at the University of Georgia. Referrals came from local clinicians (eg, psychiatrists, psychologists, social workers, and school psychiatrists) to perform diagnostic assessment and monitoring evaluations for youth displaying early psychotic symptoms. Additional recruitment methods included online and print advertisements, in-person presentations to community mental health centers, and calls or in-person meetings with members of the local school system. Clinical high-risk participants met progression or persistence criteria for at least 1 psychosis-risk syndrome on the Structured Interview for Psychosis-Risk Syndromes (SIPS)^33^ and had never met criteria for a full psychotic disorder as determined by the SCID-5. Relatives were referred to the study by the CHR participants. Clinical high-risk participants were instructed to choose a REL and provide them with the lab’s contact information. Research staff then verified their REL status after receiving their contact for inclusion. They were provided with information about the purpose of the study and incentives.

All participants were paid $30 for completing the study procedures. Clinical high-risk participants received an additional $20 for referring a REL who participated. Ten CHR and 5 REL also completed an additional qualitative interview conducted over zoom. They received an additional $30/hour for their time in the qualitative interview. All participants were paid via printed check. The study was approved by the University of Georgia Institutional Review Board.

The demographic profiles of the CHR and REL groups were generally similar (see Table 1).

**Table 1:** Participant Demographics

	CHR (*n* = 45)	REL (*n* = 18)
**Age; *M* (SD)**	25.62 (4.33)	33.11 (16.62)
**% Male**	33.3%	50.00%
**Personal education**	15.16 (2.48)	14.50 (2.46)
**Parental education**	14.80 (3.03)	12.97 (3.41)
**Race**	—	—
**Black**	6.70%	5.60%
**Biracial**	8.90%	0.00%
**White**	66.70%	83.30%
**Hispanic/Latinx**	8.90%	4.50%
**Asian**	8.90%	11.10%
**REL relationship to CHR subject**	—	—
**Sibling**	—	38.90%
**Partner**	—	38.90%
**Parent**	—	22.20%
**Years known**	—	17.67 (10.92)
** Sibling**	—	22.71 (4.27)
** Partner**	—	6.00 (4.32)
** Parent**	—	29.25 (6.75)
**NSI-PR total score**	14.02 (6.88)	—

Abbreviations: CHR, clinical high risk for psychosis; REL, relative.

### Procedures

Clinical high-risk participants were administered the NSI-PR^9^ by research staff to assess negative symptom severity. Research staff were trained to reliability standards using the gold-standard NSI-PR training materials. To be eligible, CHR participants had to have at least one NSI-PR item rated 2 (mild) or higher.

Participants were instructed to complete a structured survey over the Qualtrics website on their own. The survey contained questions designed to evaluate their self-perceptions of the use of digital phenotyping in assessing negative symptoms. An analogous survey was given to REL.

The full survey is provided in Supplemental Materials. Participants were initially presented with descriptions of 6 digital phenotyping technologies: (1) Geolocation: Phone GPS measures behaviors such as leaving the house; visiting more locations throughout the day; traveling a greater distance throughout the day; (2) Accelerometry: smart bands or phone sensors measure movement throughout the day; (3) VOX: the phone microphone measures the presence of speech, which can indicate more social interaction, more talking throughout the day, and greater changes in emotional tone in speech; (4) Ambulatory video: selfie videos, audio, or photo recordings taken by phone measure emotional changes in facial expression, voice, and body gestures; (5) Turn latency: phone audio recordings of speech measure response speed after someone asks a question; and (6) EMA surveys: phone surveys delivered multiple times per day asking about behaviors such as activities, social interactions, and pleasurable emotional experiences. They were asked about their rating of each of the 6 digital phenotyping technologies in relation to: general importance, importance for social function, importance for occupational function, and quality of life. The rating was presented on a scale from 0 to 3 (0 = not at all, 1 = slightly, 2 = somewhat, and 3 = extremely). Next, participants were asked to give their rating on the same scale (0-3) about how concerned they were about using digital phenotyping to measure their negative symptoms, followed by a prompt to explain what their concerns were. Finally, they were asked how well they thought each of the technologies could measure their negative symptoms on the same scale (0-3).

Lastly, participants were presented with our standard list of EMA questions that we have used in several past studies to measure negative symptoms and were asked to (1) evaluate if the questions were clear; (2) indicate what they thought each question was asking about if it was unclear.^14^^,^^15^^,^^21^^,^^34–38^

After participants completed the survey, qualitative interviews were administered to a subset of CHR (*n* = 10) and REL (*n* = 5) participants to provide more detailed explanations on the responses received on the Qualtrics surveys. The qualitative interview is presented in the Supplementary Material.

The number of CHR participants who completed prior EMA/Digital phenotyping studies was *n* = 38. Protocols they completed focused on emotion regulation or negative symptoms, and included some EMA items and passive methods similar to those queried in the current study.^28^^,^^29^^,^^34^^,^^39–43^

### Data Analysis

Qualitative and quantitative data were collected as complementary but independent parts of this study to characterize perceptions of digital phenotyping measures. The qualitative data were not intended for methodological triangulation; rather, they were collected to provide additional contextual information regarding participants’ perspectives of acceptability.

Descriptive statistics were calculated separately for CHR and REL participants for each quantifiable question (ie, yes/no and ordinal ratings).

For qualitative data, we used a coding reliability thematic analysis approach. A structured codebook was developed. Two independent coders applied codes to evaluate consistency among coding and disagreements were resolved through adjudication. Coding frameworks were developed after 2 sets of coders took a first pass at reviewing the transcriptions for each question. Analytic decisions regarding themes were discussed in team meetings, and structured disagreement discussions helped to consider alternative interpretations and challenge assumptions.

Additionally, supplemental analyses were conducted on the quantitative and qualitative data, comparing individuals at CHR with and without past exposure to digital phenotyping methods.

## Results

Negative symptom severity of CHR participants is reported in Table 1 (*M* = 14.02, SD = 6.88). Descriptive statistics are provided in Table 2 for CHR and REL participants for subjective perceptions of all 6 technologies in relation to: general importance, social function, occupational function, and quality of life. As can be seen in Table 2, the highest general importance rating was given to behaviors measured by EMA surveys and accelerometry, with the lowest importance given to ambulatory videos. Regarding the importance of the behaviors measured by the technologies on work function, EMA surveys, geolocation, and VOX received the highest ratings, while ambulatory videos received the lowest. For social functioning, EMA surveys and VOX received the highest ratings and turn latency was the lowest. For quality of life, EMA surveys, geolocation, and accelerometry were deemed most important, whereas ambulatory videos and turn latency were lower.

**Table 2:** Participant Rating of Behaviors Measured by Digital Phenotyping Technologies

Digital phenotyping technology	CHR: *M* (SD)				REL: *M* (SD)			
	Importance	Work functioning	Social functioning	Quality of life	Importance	Work functioning	Social functioning	Quality of life
Leaving the house; visiting more locations throughout the day; traveling a greater distance throughout the day (measured with phone GPS)	1.44 (0.97)	1.51 (1.14)	1.93 (1.03)	2.20 (0.85)	1.33 (0.97)	1.67 (1.28)	1.61 (1.09)	1.94 (1.26)
Moving more throughout the day (measured with a smartband or phone sensors)	2.20 (0.92)	1.73 (1.07)	1.60 (1.03)	2.53 (0.76)	1.61 (1.09)	1.50 (1.15)	1.44 (1.15)	2.18 (1.07)
More social interactions; talking more throughout the day; more changes in emotional tone in your speech (measured with a phone microphone that can detect when speech happens)	1.50 (1.00)	1.98 (1.01)	2.29 (0.99)	2.02 (0.93)	1.39 (1.09)	1.44 (1.38)	1.78 (1.22)	1.83 (1.20)
Making more emotional changes in your face, voice, and body gestures (measured with selfie video, audio, or photo recordings you would take from your phone to answer questions about your symptoms)	1.02 (0.95)	1.30 (0.98)	1.60 (1.01)	1.13 (0.97)	0.78 (0.94)	1.00 (1.03)	1.41 (1.18)	1.33 (1.14)
Responding faster after someone asks you a question (measured with audio recordings of your speech)	1.20 (0.95)	1.62 (1.07)	1.49 (1.04)	1.38 (1.15)	1.06 (1.00)	1.17 (1.25)	1.18 (1.19)	1.33 (1.24)
Having more activities, social interactions, and pleasurable emotional experiences (measured with surveys you would complete on your phone multiple times per day)	2.00 (0.94)	1.96 (0.90)	2.13 (0.97)	2.42 (0.94)	1.72 (0.90)	1.67 (1.28)	1.67 (0.97)	2.00 (1.14)

Abbreviations: CHR, clinical high risk for psychosis; REL, relative

Table 3 presents descriptive statistics on how concerned CHR and REL participants were about using digital phenotyping technology to measure their symptoms, as well as representative qualitative explanations of those concerns provided in the Qualtrics survey (additional quotations can be found in Table S1). Results indicated that the overall magnitude of concern was low across all technologies, with mean values falling around 1 (slightly concerned) for each technology. Vocal acoustic activity received the highest concern level. Qualitative comments indicated that common themes of the concerns noted by CHR participants involved: privacy issues, data security, GPS/location tracking, video/audio recordings, data validity, emotional burden, surveys being burdensome, concern with turn latency, and technology interfering with daily activities. Relatives reported similar themes in their concerns, including: privacy, data security, data validity, emotional burden, technology interfering with daily activities, and repeated surveys being burdensome.

**Table 3:** Descriptive Statistics and Example Qualitative Explanation of Participants’ Concern About Digital Phenotyping Technology

Digital phenotyping technology	CHR rating *M* (SD)	REL rating *M* (SD)	CHR theme and quote	REL theme/quote
Leaving the house; visiting more locations throughout the day; traveling a greater distance throughout the day (measured with phone GPS)	0.80 (1.04)	0.39 (0.70)	**Privacy concerns** “Privacy concerns especially as it relates to answering questions and conversations” **Data security** “I worry about info like location and audio being tracked and used by organizations to target ads and sell data.” **GPS/location tracking** “I don’t like the idea of being digitally monitored, especially location. It can feel like an invasion of privacy.” **Data validity** “Watching myself react / respond can stir up insecurities and make it more challenging to respond genuinely.” **Emotional burden** “Consistently recording/analyzing one’s speech/mannerisms/social interaction may cause more negative symptoms…” **Surveys being burdensome** “Time commitment of the survey” “My only concern with the surveys is they would be a nuisance and I would not keep up with them.” **Turn latency** “I just don’t think I would love the idea of an app that is forever timing how long it takes me to respond when in conversation” **Interfering with daily activities** “Spending so much time on my phone would take me out of the moment and have me preoccupied on improving my ‘score.’” “Too much time on the phone”	**Privacy concerns** “I distrust technology and people ability to keep others from abusing it.” **Data security** “Data storage and security of technology features.” **Emotional burden** “It could also make it feel more like a test to achieve these goals or you will fail and thus feel worse. A similar thing happened with apple watch activity tracker. People got obsessed with filling the rings and would feel bad about themselves if they failed to do so.” **Technology interfering with daily activities** “Just concerned that she would rather sit in her phone all day then do anything.” **Data validity** “I don’t believe there’s a correlation between distance traveled and lack of a negative symptom. If people know they’re being listened to via microphone, you might get less accurate responses.” **Repeated surveys being burdensome** “I am a little concerned about the surveys being burdensome due to other responsibilities, but not too worried.”
Moving more throughout the day (measured with a smartband or phone sensors)	0.40 (0.72)	0.22 (0.73)		
More social interactions; talking more throughout the day; more changes in emotional tone in your speech (measured with a phone microphone that can detect when speech happens)	1.67 (1.30)	1.06 (1.11)		
Making more emotional changes in your face, voice, and body gestures (measured with selfie video, audio, or photo recordings)	1.22 (1.24)	0.61 (0.92)		
Responding faster after someone asks you a question (measured with audio recordings of speech)	1.29 (1.25)	0.67 (0.97)		
Having more activities, social interactions, and pleasurable emotional experiences (measured with surveys you would complete on your phone multiple times per day)	0.62 (0.96)	0.39 (0.85)		

Abbreviations: CHR, clinical high risk for psychosis; REL, relative.

Results from the full qualitative interviews provided further insight into participants’ concerns and trust of digital phenotyping technologies (see Tables S2 and S3). Some notable themes of CHR participants included: concerns and misunderstandings about voice detection, trust and comfort with geolocation, and some discomfort with recording videos. In REL participants, some common themes included trust and comfort with geolocation, accelerometry, and video/audio recordings, and receiving more information on how data are collected and stored would ease their concerns. Both groups reported active digital phenotyping measures such as EMA surveys to be acceptable but time-consuming and inconvenient at times and felt reassured when speech detection was explained in detail. They also highlighted the importance of the technologies being noninvasive, having control over turning data collection on/off, and the data being safely stored.

Table 4 presents descriptive statistics for CHR and REL participants regarding subjective perceptions of how well each of the 6 technologies measure their negative symptoms. Participants in both groups thought behaviors measured by smartphones or smartbands would moderately measure their negative symptoms, with the highest ratings given to accelerometry and the lowest for ambulatory videos.

**Table 4:** Participant Rating of How Well Negative Symptoms Are Measured by Digital Phenotyping Technologies

Digital phenotyping technology	CHR: *M* (SD)	REL: *M* (SD)
Leaving the house; visiting more locations throughout the day; traveling a greater distance throughout the day (measured with phone GPS)	1.96 (0.90)	1.56 (1.04)
Moving more throughout the day (measured with a smartband or phone sensors)	2.16 (0.82)	2.06 (1.00)
More social interactions; talking more throughout the day; more changes in emotional tone in your speech (measured with a phone microphone that can detect when speech happens)	1.53 (0.84)	1.28 (0.90)
Making more emotional changes in your face, voice, and body gestures (measured with selfie video, audio, or photo recordings you would take from your phone to answer questions about your symptoms)	1.11 (0.86)	0.94 (0.94)
Responding faster after someone asks you a question (measured with audio recordings of your speech)	1.22 (0.77)	1.22 (1.06)
Having more activities, social interactions, and pleasurable emotional experiences (measured with surveys you would complete on your phone multiple times per day)	1.91 (0.87)	1.78 (1.00)

Abbreviations: CHR, clinical high risk for psychosis; REL, relative.

Participants viewed our standard list of EMA questions used to measure negative symptoms and indicated that the questions were clear at 95.8% for CHR participants and 100% for REL participants. Qualitative responses were provided in instances where EMA questions were found unclear (see Table 5). Responses noted instances where EMA items were too vague or difficult to understand. Participants provided helpful suggestions for wording such items (see Table S6).

**Table 5:** Participant Rating and Qualitative Explanation of EMA Surveys

	CHR			REL
EMA questions	Rating	Explanation	Rating	Explanation
Where are you? / What are you doing?	98%	“They’re fine but maybe smoking could be changed to ‘using substances’? Maybe or maybe put another category.”	100%	-
How interested are you in what you’re doing? / How motivated are you to do what you’re doing? / How much are you enjoying what you’re doing? / How much do you think you will enjoy what you’re doing the next time you do it? / How much do you want to be doing something right now?	89%	“That last one, is it supposed to be related? If it is part of a series of question and kind of threw me off, and I don’t understand the relation. If it’s on its own, then it’s fine. Personally, I don’t like that last one. I feel like it’s pretty vague.” “If they’re filling out the survey they may think that’s what they’re doing. Kind of confused what we’re answering about.” “The difference between the first three isn’t totally clear, and I don’t quite understand the last question. Like as opposed to doing nothing?”	100%	-
Who are you talking to or spending your time with?	98%	“Is this asking about right now, this week, on a regular basis or what? They’re not actively talking to someone as they fill out the survey so this could be confusing.”	100%	-
How are you talking to them?	98%	“If you’re doing a survey, you’re probably not talking to anyone. It needs another option maybe?” “Kind of vague, I imagine it is asking me how I am talking to the people that I am currently spending most of my time with.”	100%	-
How interested are you in who you are talking to or spending time with? / How motivated do you feel to spend time with them? / How much are you enjoying talking to or spending time with them? / How much do you think you will enjoy talking to or spending time with them next time?	96%	“The first question may be taken as dating interest” “First three are too similar”	100%	-

Abbreviations: CHR, clinical high risk for psychosis; EMA, ecological momentary assessment; REL, relative.

Results from supplemental analyses showed that key quantitative and qualitative variables did not differ between CHR participants with (*n* = 38) and without (*n* = 7) previous exposure to digital phenotyping measures.

## Discussion

The assessment of negative symptoms is crucial for the early identification and prevention of psychosis. The development of digital phenotyping technologies over time has introduced a wide range of modalities to integrate the measurement of negative symptoms into daily life. However, little is known about how CHR and REL participants perceive the use of digital technologies. The current study therefore examined the subjective perceptions of CHR and REL participants on digital phenotyping methods for assessing negative symptoms. Several important findings emerged.

First, both groups had minimal concerns about using digital phenotyping. The average numerical value they assigned to each technology was approximately “slightly” concerned. Both groups expressed low concerns about continuous GPS tracking, smartphones/smartbands tracking movements via accelerometry, and EMA surveys. The highest level of concern was given to VOX (speech detection) in both groups. Concerns with VOX mostly centered around privacy as a result of misunderstanding that the technology would record and save their conversations as they speak. Interviewers then conducted follow-up qualitative interviews with those who expressed concerns and clarified that VOX only detects the presence of speech as opposed to recording speech samples, and these explanations mitigated their concerns. A few participants reported some residual discomfort with the concept of the phone listening in the background. Additionally, presenting participants with excerpts from informed consent documents clarifying data security, how audio/video recordings will be used, and the options to turn on/off each technology helped reduce concerns CHR and REL participants had. Notably, REL participants consistently had fewer concerns than CHR participants for all technologies, indicating a higher level of openness and insight with digital phenotyping. Although CHR and REL concerns were minimal, it will of course be important to maintain best practices regarding digital phenotyping data storage (eg, SSL encryption for data in transit, storing sensitive information on users’ phones until they are in Wi-Fi/cell range and then pushing to secure servers, and length of data retention) and data sharing (eg, anonymizing, providing summary statistics instead of raw data) to ensure that participant protection is adequate.

Second, both groups rated each digital phenotyping technology as somewhat important in terms of general importance, relation to work and social functioning, and quality of life. These findings suggest that CHR and REL participants believe in the relevance of negative symptoms to their daily life and perceive value in using digital phenotyping methods to assess them. From qualitative interview results, CHR and REL participants both affirmed the benefits of digital technologies, agreeing that they could capture meaningful changes in their symptoms, such as the number of locations visited, activity level, amount of speech, and facial, voice, or body gestures.

Third, CHR and REL participants believe that digital phenotyping technologies measure negative symptoms somewhat well. Among all digital phenotyping technologies, actigraphy and accelerometry received the highest rating, suggesting that both groups viewed that one’s movement and activity level are strong indicators of the amount of goal-directed, work, or social activities they perform. This finding is promising because these objective measurement tools can be obtained passively, which makes them more feasible as no direct initiation by participants is needed. Ecological momentary assessment surveys also received high ratings in both groups, suggesting that surveys were believed to capture negative symptoms accurately.

Finally, both groups judged the EMA survey items as clear and understandable (>89% in CHR, 100% in REL). CHR participants noted that certain questions were vague and provided some suggestions for improvement, such as changing “smoking” to “using substances” and word choice being redundant.

These findings provide valuable insight into how CHR and REL participants perceive digital phenotyping methods and their concerns about them. Findings suggest that digital phenotyping methods are generally trusted, considered to measure relevant life activities, somewhat perceived by participants as accurate for assessing negative symptoms, and met with minimal concerns that were alleviated with additional clarification and details. The current study primarily focused on acceptability, clarity, burden, and perceived relevance of digital phenotyping approaches. Results may be informative for the Food and Drug Administration (FDA) Clinical Outcome Assessment (COA) qualification criterion that the concept of interest is adequately covered by the method. However, additional work may be needed specifically related to domain coverage and content validity to tie information to the 5 negative symptom domains.

There are a few limitations of the current study. First, although the two-tiered process of collecting quantitative and qualitative data helped with obtaining more comprehensive results, the follow-up qualitative interviews were not administered to all CHR and REL participants, which potentially limits the generalizability of the qualitative results. Relatedly, the REL group was small and heterogeneous in relative status, making their aggregate estimates less stable. The second limitation is that the CHR sample consisted of a mixture of clinical help-seeking cases and individuals who responded to online recruitment ads, as well as a range of ages, different degrees of comfort with technology, and access to phones/smartbands. Although those with and without prior study exposure to digital phenotyping methods did not appear to markedly differ in quantitative or qualitative responses (see Tables S4 and S5), prior exposure remains a potential source of sample heterogeneity that could potentially influence acceptability in CHR individuals. Future clinical trials that use digital phenotyping outcome measures could consider prior exposure to these measures as a relevant inclusion factor. Third, there are many other digital phenotyping technologies that were not evaluated in the current study. Those selected included frequently implemented passive and active measures, particularly in the measurement of negative symptoms. Future studies can overcome the current limitation by focusing on obtaining data on additional novel measures, such as screentime, number of nearby Bluetooth devices, texting and phone logs, key stroke data, and app usage. Finally, findings may primarily reflect the perceptions of research-engaged CHR participants and their relatives and may therefore not be representative of the entire CHR population.

## Supplementary Material

Supplementary_material_sgag034
