# Supplementary material for: Subjective Perceptions of Digital Phenotyping Measures of Negative Symptoms Among Individuals At Clinical High Risk for Psychosis and Their Relatives
Source: Schizophr Bull Open. 2026 Aug 18;7(1):sgag034. doi: 10.1093/schizbullopen/sgag034 (PMC13570644; doi:10.1093/schizbullopen/sgag034)
Supplement: Supplementary file 1 [file supplementary_material_sgag034.zip › Supplementary Materials_Digital Assessments Block.docx]

Supplemental Materials

Qualtrics Survey- Digital Assessments Block

Part 6 of 7	**Digital Assessments** We’re creating new assessments of negative symptoms using smartphones and smartbands. We’d like to get your opinion on which types of technology might be most meaningful.

1. How important is it to you to be doing well or improve on each of these behaviors measured by smartphones or smartbands?

|  | 0 Not at all | 1 Slightly | 2 Moderately | 3 Extremely |
| --- | --- | --- | --- | --- |
| 1A. Leaving the house; visiting more locations throughout the day; traveling a greater distance throughout the day (Measured with phone GPS) |  |  |  |  |
| 1B. Moving more throughout the day (Measured with a smartband or phone sensors) |  |  |  |  |
| 1C. More social interactions; talking more throughout the day; more changes in emotional tone in your speech (Measured with a phone microphone that can detect when speech happens) |  |  |  |  |
| 1D. Making more emotional changes in your face, voice, and body gestures (Measured with selfie video, audio, or photo recordings you would take from your phone to answer questions about your symptoms) |  |  |  |  |
| 1E. Responding faster after someone asks you a question (measured with audio recordings of your speech) |  |  |  |  |
| 1F. Having more activities, social interactions, and pleasurable emotional experiences (Measured with surveys you would complete on your phone multiple times per day) |  |  |  |  |

1. How important is it to you to be doing well or improve on each of these behaviors measured by smartphones or smartbands?

|  | 0 Not at all | 1 Slightly | 2 Moderately | 3 Extremely |
| --- | --- | --- | --- | --- |
| 1A. Leaving the house; visiting more locations throughout the day; traveling a greater distance throughout the day (Measured with phone GPS) |  |  |  |  |
| 1B. Moving more throughout the day (Measured with a smartband or phone sensors) |  |  |  |  |
| 1C. More social interactions; talking more throughout the day; more changes in emotional tone in your speech (Measured with a phone microphone that can detect when speech happens) |  |  |  |  |
| 1D. Making more emotional changes in your face, voice, and body gestures (Measured with selfie video, audio, or photo recordings you would take from your phone to answer questions about your symptoms) |  |  |  |  |
| 1E. Responding faster after someone asks you a question (measured with audio recordings of your speech) |  |  |  |  |
| 1F. Having more activities, social interactions, and pleasurable emotional experiences (Measured with surveys you would complete on your phone multiple times per day) |  |  |  |  |

2. How much would doing well on each of the following make it easier for you to have a job or go to school?

|  | 0 Not at all | 1 Slightly | 2 Moderately | 3 Extremely |
| --- | --- | --- | --- | --- |
| 2A. Leaving the house; visiting more locations throughout the day; traveling a greater distance throughout the day (Measured with phone GPS) |  |  |  |  |
| 2B. Moving more throughout the day (Measured with a smartband or phone sensors) |  |  |  |  |
| 2C. More social interactions; talking more throughout the day; more changes in emotional tone in your speech (Measured with a phone microphone that can detect when speech happens) |  |  |  |  |
| 2D. Making more emotional changes in your face, voice, and body gestures (Measured with selfie video, audio, or photo recordings you would take from your phone to answer questions about your symptoms) |  |  |  |  |
| 2E. Responding faster after someone asks you a question (measured with audio recordings of your speech) |  |  |  |  |
| 2F. Having more activities, social interactions, and pleasurable emotional experiences (Measured with surveys you would complete on your phone multiple times per day) |  |  |  |  |

3. How much would doing well on each of the following make it easier for you to have relationships with friends, family, or romantic partners?

|  | 0 Not at all | 1 Slightly | 2 Moderately | 3 Extremely |
| --- | --- | --- | --- | --- |
| 3A. Leaving the house; visiting more locations throughout the day; traveling a greater distance throughout the day (Measured with phone GPS) |  |  |  |  |
| 3B. Moving more throughout the day (Measured with a smartband or phone sensors) |  |  |  |  |
| 3C. More social interactions; talking more throughout the day; more changes in emotional tone in your speech (Measured with a phone microphone that can detect when speech happens) |  |  |  |  |
| 3D. Making more emotional changes in your face, voice, and body gestures (Measured with selfie video, audio, or photo recordings you would take from your phone to answer questions about your symptoms) |  |  |  |  |
| 3E. Responding faster after someone asks you a question (measured with audio recordings of your speech) |  |  |  |  |
| 3F. Having more activities, social interactions, and pleasurable emotional experiences (Measured with surveys you would complete on your phone multiple times per day) |  |  |  |  |

4. How much would doing well on each of the following improve the quality of your life?

|  | 0 Not at all | 1 Slightly | 2 Moderately | 3 Extremely |
| --- | --- | --- | --- | --- |
| 4A. Leaving the house; visiting more locations throughout the day; traveling a greater distance throughout the day (Measured with phone GPS) |  |  |  |  |
| 4B. Moving more throughout the day (Measured with a smartband or phone sensors) |  |  |  |  |
| 4C. More social interactions; talking more throughout the day; more changes in emotional tone in your speech (Measured with a phone microphone that can detect when speech happens) |  |  |  |  |
| 4D. Making more emotional changes in your face, voice, and body gestures (Measured with selfie video, audio, or photo recordings you would take from your phone to answer questions about your symptoms) |  |  |  |  |
| 4E. Responding faster after someone asks you a question (measured with audio recordings of your speech) |  |  |  |  |
| 4F. Having more activities, social interactions, and pleasurable emotional experiences (Measured with surveys you would complete on your phone multiple times per day) |  |  |  |  |

5. Do you have concerns about using any of the technology described below to measure your negative symptoms?

|  | 0 Not at all | 1 Slightly | 2 Moderately | 3 Extremely |
| --- | --- | --- | --- | --- |
| 5A. Leaving the house; visiting more locations throughout the day; traveling a greater distance throughout the day (Measured with phone GPS) |  |  |  |  |
| 5B. Moving more throughout the day (Measured with a smartband or phone sensors) |  |  |  |  |
| 5C. More social interactions; talking more throughout the day; more changes in emotional tone in your speech (Measured with a phone microphone that can detect when speech happens) |  |  |  |  |
| 5D. Making more emotional changes in your face, voice, and body gestures (Measured with selfie video, audio, or photo recordings you would take from your phone to answer questions about your symptoms) |  |  |  |  |
| 5E. Responding faster after someone asks you a question (measured with audio recordings of your speech) |  |  |  |  |
| 5F. Having more activities, social interactions, and pleasurable emotional experiences (Measured with surveys you would complete on your phone multiple times per day) |  |  |  |  |

What is the concern?

________________________________________________________________

________________________________________________________________

________________________________________________________________

________________________________________________________________

________________________________________________________________

6. How much do you think behaviors measured by smartphones or smartbands would measure your negative symptoms?

|  | 0 Not at all | 1 Slightly | 2 Moderately | 3 Extremely |
| --- | --- | --- | --- | --- |
| 6A. Leaving the house; visiting more locations throughout the day; traveling a greater distance throughout the day (Measured with phone GPS) |  |  |  |  |
| 6B. Moving more throughout the day (Measured with a smartband or phone sensors) |  |  |  |  |
| 6C. More social interactions; talking more throughout the day; more changes in emotional tone in your speech (Measured with a phone microphone that can detect when speech happens) |  |  |  |  |
| 6D. Making more emotional changes in your face, voice, and body gestures (Measured with selfie video, audio, or photo recordings you would take from your phone to answer questions about your symptoms) |  |  |  |  |
| 6E. Responding faster after someone asks you a question (measured with audio recordings of your speech) |  |  |  |  |
| 6F. Having more activities, social interactions, and pleasurable emotional experiences (Measured with surveys you would complete on your phone multiple times per day) |  |  |  |  |

Below is the survey we send to people’s phones to tell if they have negative symptoms. We’d like to get your opinion on these questions. Is each set of questions clear? If not, please explain to us what you think the question is trying to ask about.

7A.

Where are you?

- Home
- Work/School
- Friend/Family home
- Public place (bus, store, etc)
- Clinic/Doctor's office

What are you doing?

- Working/Studying
- Eating/Drinking
- Hobby/Having fun
- Internet/Computer use
- Errands/Housework
- Resting
- Exercising
- Shopping
- TV/Music
- Traveling
- Smoking
- Bathing/Washing up
- Pacing restlessly
- Spending time with someone
- Nothing

Are the questions clear?

- Yes
- No

If no, explain what you think this question was asking about in your own words:

________________________________________________________________

Below is the survey we send to people’s phones to tell if they have negative symptoms. We’d like to get your opinion on these questions. Is each set of questions clear? If not, please explain to us what you think the question is trying to ask about.

7B. Please complete the following:

- How interested are you in what you’re doing?
- How motivated are you to do what you’re doing?
- How much are you enjoying what you’re doing?
- How much do you think you will enjoy what you’re doing the next time you do it?
- How much do you want to be doing something right now?

Are the questions clear?

- Yes
- No

If no, explain what you think this question was asking about in your own words:

________________________________________________________________

Below is the survey we send to people’s phones to tell if they have negative symptoms. We’d like to get your opinion on these questions. Is each set of questions clear? If not, please explain to us what you think the question is trying to ask about.

7C. Who are you talking to or spending your time with?

- Husband/ Wife/ Boyfriend/ Girlfriend
- Family/ Roommates
- Friends Coworkers/ Classmates
- Doctor/ Therapist
- Strangers
- No one/ Alone

Are the questions clear?

- Yes
- No

If no, explain what you think this question was asking about in your own words:

________________________________________________________________

Below is the survey we send to people’s phones to tell if they have negative symptoms. We’d like to get your opinion on these questions. Is each set of questions clear? If not, please explain to us what you think the question is trying to ask about.

7D. How are you talking to them?

- In person
- Phone/ Video call
- Electronically (text, social media, email etc.)

Are the questions clear?

- Yes
- No

If no, explain what you think this question was asking about in your own words:

________________________________________________________________

Below is the survey we send to people’s phones to tell if they have negative symptoms. We’d like to get your opinion on these questions. Is each set of questions clear? If not, please explain to us what you think the question is trying to ask about.

7E. Please complete the following:

- How interested are you in who you are talking to or spending time with?
- How motivated do you feel to spend time with them?
- How much are you enjoying talking to or spending time with them?
- How much do you think you will enjoy talking to or spending time with them next time?

Are the questions clear?

- Yes
- No

If no, explain what you think this question was asking about in your own words:

________________________________________________________________

**End of Block: Digital Assessments**
