# Supplementary material for: Subjective Perceptions of Digital Phenotyping Measures of Negative Symptoms Among Individuals At Clinical High Risk for Psychosis and Their Relatives
Source: Schizophr Bull Open. 2026 Aug 18;7(1):sgag034. doi: 10.1093/schizbullopen/sgag034 (PMC13570644; doi:10.1093/schizbullopen/sgag034)
Supplement: Supplementary file 1 [file supplementary_material_sgag034.zip › Supplementary_Materials-Tables2_sgag034.docx]

Supplementary Materials

Supplementary Table 1. Full Table with descriptive statistics and qualitative explanation of participants’ concern about digital phenotyping technology

| **Digital Phenotyping Technology** | **CHR Rating**  **M (SD)** | **REL**  **Rating**  **M (SD)** | **CHR Theme & Quote** | **REL Theme/Quote** |
| --- | --- | --- | --- | --- |
| Leaving the house; visiting more locations throughout the day; traveling a greater distance throughout the day (measured with phone GPS) | 0.80 (1.04) | 0.39 (0.70) | **Privacy concerns**  “…always recording speech is extremely concerning to me because of privacy concerns”  “Privacy concerns especially as it relates to answering questions and conversations”  “I just don’t like entities, no matter who they are, listening in on private conversations of mine”  **Data Security** “I worry about info like location and audio being tracked and used by organizations to target ads and sell data.”  “Im concern about how the data collected can identify me as an individual”.  “The growing Police state, my data being sold to surveillance entities and my privacy being compromised.”  **GPS/location tracking**  “I don't like the idea of being digitally monitored, especially location. It can feel like an invasion of privacy.”  **Data validity**  “Watching myself react / respond can stir up insecurities and make it more challenging to respond genuinely.”  “how would it differentiate between me talking to someone versus watching tv or a podcast or just sitting on the bus next to people talking.”  “…I understand that making more changes in the emotional tone of your speech (for example) could make having social interactions with people easier, but I don’t think it is necessarily better to have more emotional changes in your tone of speech than to have less.”  **Emotional Burden**  “Consistently recording/analyzing one’s speech/mannerisms/social interaction may cause more negative symptoms. I think people struggling with these symptoms are more susceptible to self-deprecation and criticism/have preexisting issues with self-image and perception...I don’t think it’s necessary to watch or hear yourself back during interactions and to pick apart what you should or shouldn’t have done; I think it’d be more beneficial and more organic to observe healthy communication skills and expressions from other people via videos, real-life interactions, demonstrations, etc.”  **Surveys being burdensome**  “Time commitment of the survey”  “My only concern with the surveys is they would be a nuisance and I would not keep up with them.”  **Turn latency**  “I just don't think I would love the idea of an app that is forever timing how long it takes me to respond when in conversation”  **Interfering with daily activities**  “Spending so much time on my phone would take me out of the moment and have me preoccupied on improving my "score"”  “Too much time on the phone” | **Privacy concerns**  “I distrust technology and people ability to keep others from abusing it.”  “I am concerned about the privacy of the microphone tracking speech all day”  “Will being recorded not give them privacy?”  **Data security**  “Data storage and security of technology features.”  **Emotional burden**  “It could also make it feel more like a test to achieve these goals or you will fail and thus feel worse. A similar thing happened with apple watch activity tracker. People got obsessed with filling the rings and would feel bad about themselves if they failed to do so.”  **Technology interfering with daily activities**  “Just concerned that she would rather sit in her phone all day then do anything”.  “Will filing out surveys take away for being in the moment?”    **Data validity**  “I don't believe there’s a correlation between distance traveled and lack of a negative symptom. If people know they’re being listened to via microphone, you might get less accurate responses.”  “…the ability of it (microphone) to do so if I'm in noisy environments.”  **Repeated surveys being burdensome**  “I am a little concerned about the surveys being burdensome due to other responsibilities, but not too worried” |
| Moving more throughout the day (measured with a smartband or phone sensors) | 0.40 (0.72) | 0.22 (0.73) |  |  |
| More social interactions; talking more throughout the day; more changes in emotional tone in your speech (measured with a phone microphone that can detect when speech happens) | 1.67 (1.30) | 1.06 (1.11) |  |  |
| Making more emotional changes in your face, voice, and body gestures (measured with selfie video, audio, or photo recordings you would take from your phone to answer questions about your symptoms) | 1.22 (1.24) | 0.61 (0.92) |  |  |
| Responding faster after someone asks you a question (measured with audio recordings of your speech) | 1.29 (1.25) | 0.67 (0.97) |  |  |
| Having more activities, social interactions, and pleasurable emotional experiences (measured with surveys you would complete on your phone multiple times per day) | 0.62 (0.96) | 0.39 (0.85) |  |  |

Note. CHR = Clinical High Risk for psychosis; REL = relative

Supplementary Table 2. Themes from qualitative interviews on subjective perceptions of digital phenotyping for CHR participants

| Concerns | Themes | Quotes |
| --- | --- | --- |
| Overall Impressions | Innovative | “I mean, I think that it's… it's a really innovative thought, and it's… it could be a useful tool. maybe not practical for everybody, because there's a lot of people who don't have access, I think, still to… Like, things like wearables and things of that nature, but I think it's a really great idea.” |
| Relevance to Daily Life | Sleep patterns | “Well, I mean, with… I feel like a wearable would be really good for, like, monitoring sleep patterns, or, quality of sleep, or lack thereof, quality of sleep.” |
| Concerns: | Voice detection | “I don't have any issue with the GPS location. The only concern I may have, like, my job, we do a lot of confidential stuff, so the thought of having my phone being able to listen to” |
| GPS/Geolocation | Social functioning | “Especially with the GPS, I mean, it really shows, are you in one place all day long, not doing anything, not having relationships, not getting outside, not doing those things that are good for us mentally?” |
| Smart Bands/Accelerometry | Correlated with mood; May be uncomfortable to wear | And, like, how… My mood affects, like, my amount of movement and stuff like that.  “The phone is better for me than actual wearable devices… I’ve never been able to consistently wear a watch.” |
| Microphone/Speech Detection | Highly misunderstood by most participants thinking it’s actually recording speech; reassured by clarification | “Yes, 100%. (knowing it doesn’t record actual speech makes them feel better)”  “Yeah, the audio… yeah, the audio recordings being handled in a secure way, that worries me.”  “Just because it’s like, if it can hear what I’m speaking, that means regardless if it’s being recorded, it is in some way always recording the, you know, the ambient air, which is kind of, like, a scary thought, but I mean, I’m comfortable with it, it doesn’t really bother me.” |
| Selfie Video/Audio Recordings | Uncomfortable and can be inaccurate | “People don’t have to be truthful… super depressed people fake happiness a lot.”  “I never did [videos] because I felt insecure… I was often in public, didn’t feel comfortable videoing myself.”  “It’s a little creepy… but I understand it’s for science.” |
| Speech Turn Latency | Acceptable, could be better explained | “I think it might be useful.”  “I have conversation paralysis… a number to show me would help.”  “That's probably okay, but I think… it goes into about how it’s collected” |
| EMA Surveys | Acceptable; Time-consuming | “I don't have a problem with doing it, but it would definitely be something that would be hard to incorporate into the current life I’m living”  “It was kind of difficult… because I have work and it’s hard to stop what you’re doing completely.”  “Five minutes doesn’t seem long, but during an active workday… I’d get halfway through and someone would come talk to me.” |
| Privacy and Data Security | Better explanation about how the data is collected and stored | “As long as I consent to being tracked, I really don’t care… I feel relatively confident that the information is safe.”  “Maybe information about how the data is being protected.”  “You just never know who could get their hands on that type of material… it could be used for a wide range of things that I don’t know.”  “A nefarious hacker getting in … sell the data to people who want to use it in a bad way.” |
| Burden and Convenience | Invasive | I wouldn't say burdensome, but it does feel a little bit invasive, I'd say, maybe? I think the populations you work with struggle with trust. |
| Control and Flexibility | Necessary | “It’s nice to have the choice [to turn off [recordings] … that is a highly important aspect.”  “Having the option to turn it off while I was at work… that would make me feel better.” |
| Trust and Validity | Mixed, trustworthy but also skeptical | “If you are leaving the house more… it says something about you being more productive.”  “I have trust if you say it’s safe.”  “Why is it necessary when you already ask about social interaction?” |
| Advice/Suggestions | Different suggestions | “I'd say it's just, it's very varied data based on what the person, like, experiences… someone like me who, like, works, like, sometimes you could just be getting very like, brief and not all-encompassing answers, because, like, I was just trying to get them done, like, while the window was open, versus, like, someone who may be, like, in college, who's, like, has time to do those types of things…”  “I’d want to know that the data is helping.”  “Give a little bit of caution that we still are people that deserve privacy. Even if we are consenting to care, we are still humans.” |

Supplementary Table 3. Themes from qualitative interviews on subjective perceptions of digital phenotyping for REL participants

| Concerns | Themes | Quotes |
| --- | --- | --- |
| Overall Impressions | Acceptable, until a certain limit | “It’s fine in spurts… I wouldn’t want to be tracked every second of every day by someone.”  “She wears an Apple Watch… she’s pretty in tune with all that.”  “There's a… there's… it can be helpful, but there's a… there's a line before it's being abused.” |
| Relevance to Daily Life | Motivation | “Because if you're wanting to see if she's motivated. you can see where she's going. So you can see if she's going to the gym, or is she going for a nice long walk at the park? Like, you can see these things, or is she just sitting at home.” |
| Concerns: | Data storing | “But, you know, be like, yeah, I'd rather not… I'd rather… The less of my data is out there is the better.” |
| GPS/Geolocation | Increased communication, but comfortable | “As long as you tell people… before you do it, and you give them the option to not, I think that's fine. “ |
| Smart Bands/Accelerometry | Well accepted | “Again, if she's fine with it, I'm fine with it.”  “They already use a smartwatch to track steps.”  “Activity level reflects their symptoms — very much.” |
| Microphone/Speech Detection | Reassured by clarification, doubtful about accuracy | “I think they would be fine with that”  “Mostly when I don't really know how it works, but, in terms of, like, how the technology is actually working, and what they're, like, the process they use to differentiate between the things, but, I guess it's, like, how… Does being in a very crowded area, you know How do they know the difference?”  “Before… I wasn’t aware that it was not actually recording… now it’s fine.”  “Knowing this (not recording words) makes it more acceptable.”  “How does it know it’s speech, not TV?” |
| Selfie Video/Audio Recordings | Acceptable | “I feel like they would be very neutral about it” |
| Speech Turn Latency | Some endorsement | “Yes, it relates to their symptoms.”  “For them specifically? No, I don’t think so.” |
| EMA Surveys | Time-consuming | “I’m fine with it as long as it doesn’t affect social relationships or their job”  “If she could say what time for the things to come through… that would make it easier.”  “The randomness might be a little stressful given their full-time job.” |
| Privacy and Data Security | Fears around voice recording | “They wouldn't want the voice thing on.”  “Be careful at not being too intrusive.”  “What if there’s a data breach, and their personal information gets out?” |
| Burden and Convenience | Can become inconvenient | “It might be very difficult to… step out and record a video.”  “If they feel like they have an obligation toward something, it would stress them out if they couldn’t get to it.”  “I don’t think it would be too interruptive… depends on how long they went for.” |
| Control and Flexibility | Important to some | “I think it is important to them” |
| Trust and Validity | Not full trust in measurement devices | Then the… like, I know that, like, step recorders aren't extremely accurate, but your… your goal is a little different, so it seems like it would be easier for the technology to measure.” |
| Advice/Suggestions | Explaining the study more fully; non-invasive; financial incentive | “I think just explaining what's being looked for, and what's not being looked for.”  “Some financial incentive… would make it more of a priority.”  “As long as it doesn’t affect social relationships, or put their job in danger.”  “Remember that they’re a subject, but also still a person.” |

Supplementary Table 4. ANOVAS between CHR with and without previous digital phenotyping (DP) exposure

|  | CHR without DP exposure (N = 38); M (SD) | CHR with DP exposure (N = 7); M (SD) | Test Statistics | *p* |
| --- | --- | --- | --- | --- |
| Importance | 1.86 (0.71) | 1.50 (0.61) | F (1,43) = 1.96 | .169 |
| Work functioning | 1.81 (0.60) | 1.66 (0.78) | F (1,43) = 0.24 | .625 |
| Social functioning | 2.12 (0.62) | 1.79 (0.79) | F (1,43) = 1.10 | .300 |
| Quality of life | 2.19 (0.54) | 1.91 (0.73) | F (1,43) = 0.95 | .335 |
| Level of concerns | 1.05 (1.01) | 0.99 (0.82) | F (1,43) = 0.03 | .872 |
| How well negative symptoms are measured by DP | 1.93 (0.55) | 1.60 (0.56) | F (1,43) = 2.11 | .154 |

Supplementary Table 5. Example quotes of concerns for digital phenotyping by CHR with and without previous exposure

| CHR without digital phenotyping exposure | CHR with digital phenotyping exposure |
| --- | --- |
| 1. “Spending so much time on my phone would take me out of the moment and have me preoccupied on improving my "score”” 2. “I worry about info like location and audio being tracked and used by organizations to target ads and sell data.” 3. “I don't like the idea of being digitally monitored, especially location. It can feel like an invasion of privacy.” 4. “Struggles with self image/self perception of recording video/audio of myself” | 1. “Privacy and confidentiality concerns” 2. “I don't want to feel like my phone is listening to me” 3. “I would worry about being recorded all the time for privacy reasons. My only concern with the surveys is they would be a nuisance and I would not keep up with them.” 4. “Not a fan of the video idea. Time commitment of the survey” 5. “I often feel like I am being watched/tracked when in public.” 6. “I just don't think I would love the idea of an app that is forever timing how long it takes me to respond when in conversation” 7. “I’m concern about how the data collected can identify me as an individual” |

Supplementary Table 6. Questions and Suggestions from CHR and REL for unclear EMA survey questions

| Sample Questions | CHR | REL |
| --- | --- | --- |
| Where are you?   - Home - Work/School - Friend/Family home - Public place (bus, store, etc) - Clinic/Doctor's office   What are you doing?   - Working/Studying - Eating/Drinking - Hobby/Having fun - Internet/Computer use - Errands/Housework - Resting - Exercising - Shopping - TV/Music - Traveling - Smoking - Bathing/Washing up - Pacing restlessly - Spending time with someone - Nothing | “They’re fine but maybe smoking could be changed to “using substances”? Maybe or maybe put another category” | None |
| - How interested are you in what you’re doing? - How motivated are you to do what you’re doing? - How much are you enjoying what you’re doing? - How much do you think you will enjoy what you’re doing the next time you do it? - How much do you want to be doing something right now? | “The last one should have a scale”  “That last one, is it supposed to be related? If it is part of a series of question and kind of threw me off, and I don’t understand the relation. If it’s on its own, then it’s fine. Personally, I don’t like that last one. I feel like it’s pretty vague.”  “It seems to be asking about motivation, but some of the word choice is so weird and redundant.”  “If they’re filling out the survey they may think that’s what they’re doing. Kind of confused what we’re answering about.”  “The difference between the first three isn’t totally clear, and I don’t quite understand the last question. Like as opposed to doing nothing?” |  |
| Who are you talking to or spending your time with?   - Husband/ Wife/ Boyfriend/ Girlfriend - Family/ Roommates - Friends Coworkers/ Classmates - Doctor/ Therapist - Strangers - No one/ Alone | “Is this asking about right now, this week, on a regular basis or what? They’re not actively talking to someone as they fill out the survey so this could be confusing.” |  |
| How are you talking to them?   - In person - Phone/ Video call - Electronically (text, social media, email etc.) | “Kind of vague, I imagine it is asking me how I am talking to the people that I am currently spending most of my time with.”  “If you're doing a survey, you're probably not talking to anyone. It needs another option maybe?” |  |
| - How interested are you in who you are talking to or spending time with? - How motivated do you feel to spend time with them? - How much are you enjoying talking to or spending time with them? - How much do you think you will enjoy talking to or spending time with them next time? | “The first question may be taken as dating interest”  “First three are too similar” |  |
